# Methylomics and cancer: the current state of methylation profiling and marker development for clinical care

**DOI:** 10.1186/s12935-023-03074-7

**Published:** 2023-10-16

**Authors:** Chengyin Liu, Han Tang, Nana Hu, Tianbao Li

**Affiliations:** 1 Department of Biochemistry and Molecular & Cellular Biology, Georgetown University Medical Center, Georgetown University, Washington, DC USA; 2 BioChain (Beijing) Science & Technology Inc., Beijing, People’s Republic of China; 3 Department of Molecular Medicine, The University of Texas Health, San Antonio, USA

**Keywords:** Epigenetics, Methylation, Cancer, Detection techniques, Biomarkers

## Abstract

Epigenetic modifications have long been recognized as an essential level in transcriptional regulation linking behavior and environmental conditions or stimuli with biological processes and disease development. Among them, methylation is the most abundant of these reversible epigenetic marks, predominantly occurring on DNA, RNA, and histones. Methylation modification is intimately involved in regulating gene transcription and cell differentiation, while aberrant methylation status has been linked with cancer development in several malignancies. Early detection and precise restoration of dysregulated methylation form the basis for several epigenetics-based therapeutic strategies. In this review, we summarize the current basic understanding of the regulation and mechanisms responsible for methylation modification and cover several cutting-edge research techniques for detecting methylation across the genome and transcriptome. We then explore recent advances in clinical diagnostic applications of methylation markers of various cancers and address the current state and future prospects of methylation modifications in therapies for different diseases, especially comparing pharmacological methylase/demethylase inhibitors with the CRISPRoff/on methylation editing systems. This review thus provides a resource for understanding the emerging role of epigenetic methylation in cancer, the use of methylation-based biomarkers in cancer detection, and novel methylation-targeted drugs.

## Introduction

The term “epigenetics” was first coined in 1942 by Conrad Waddington to describe the study of phenotypic changes independent of genotypic differences in various biological systems. More specifically, epigenetics refers to heritable and potentially reversible alterations in gene expression that do not change nucleotide sequences in the genome [1, 2]. The most common mechanisms mediating epigenetic regulation include DNA methylation, regulation of non-coding RNAs, and histone modification, among which, DNA methylation was identified first and is the most well-studied [3, 4]. In 1948, Rollin Hotchkiss of the Rockefeller Institute for Medical Research first discovered cytosine modifications. Subsequently, Gerard Wyatt identified 5-methylcytosine (5mC) marks on both animal and plant DNAs, representing the first DNA methylation type reported in eukaryotes [5]. These 5mC marks are mainly located upstream of guanine (G) in the DNA double-helix at so-called CpG sites. CpG methylation alters the geometric, mechanical, and physicochemical properties of DNA, thereby affecting critical molecular processes such as DNA transcription, replication, and chromatin remodeling [6]. This breakthrough discovery of 5mC led to the identification of other methylation types, such as *N*6-methyladenosine (m^6^A) methylation, histone H3 lysine K4 (H3K4), and H3 lysine K9 (H3K9). The transcriptional regulatory effects of these modifications have been shown to play essential roles in numerous biological processes, such as genomic imprinting, cell proliferation and differentiation, and embryonic development.

The process of carcinogenesis is strongly associated with various molecular changes, such as genomic instability, epigenetic modifications, transcriptomic alterations, and post-translational modifications [7–10]. The alteration of driver genes through DNA nucleotide changes has long been recognized as an enabling characteristic in cancer progression [11]. Furthermore, dysregulation of methylation, which plays a significant role in gene expression, is observed in cancer and contributes to the development of carcinogenesis [12]. A comprehensive study examining 4302 tumors across 18 different types of cancer has demonstrated that driver gene mutations are intrinsically linked to the abnormal DNA methylation status, and these driver gene-associated methylation patterns can effectively categorize heterogeneous tumors into more homogeneous subtypes [13]. Additionally, Saghafinia et al. [14] conducted a study on a group of 126 patients with Wilms tumors to explore the potential role of aberrant methylation in pediatric tumors. Their findings confirmed that aberrant methylation patterns could serve as a hallmark of cancer in biological stages with relatively low mutation burdens. The cancer genome and epigenome are intricately interconnected in the development of oncogenic characteristics, with disruption of methylation patterns considered a crucial factor in tumorigenesis [15, 16].

Research attention has more recently focused on the contribution of DNA methylation to the pathogenesis and development of various diseases. Aberrant methylation activity has been linked to the etiology of many diseases, including cancers, cardiovascular diseases, and autoimmune disorders [17]. Abnormal methylation patterns are ubiquitously found in different cancers, with a characteristic reduction in global methylation and concomitant increase in local methylation levels [18]. By comparing differential, allele-specific methylation patterns between normal and tumor tissues, including myeloma, B-cell lymphoma, and glioblastoma, Do et al. [19] found that aberrant DNA methylation is a leading risk factor in some cancers and other non-communicable diseases. Furthermore, DNA methylation could be used to accurately distinguish different histological stages in the development of hepatocellular carcinoma (HCC), thus demonstrating the potential value of these modifications in disease monitoring in clinic [20]. The m^6^A modification is a well-established regulatory mechanism driving tumor progression [21]. For instance, Pan et al. [22] found that the RNA methyltransferase, METTL3, was highly expressed in colorectal cancer (CRC) and closely associated with overall survival and prognosis. In addition, recent work by Hogg et al. [23] uncovered links between epigenetic modifications and immunological status in the tumor microenvironment by analyzing the roles of histone acetylation and methylation in tumorigenesis and immunogenicity, and proposed therapeutic strategies targeting epigenetic regulation. Methylation thus plays an essential role in tumorigenesis and disease progression, and a comprehensive understanding of methylation mechanisms can greatly facilitate advances in targeted cancer treatment options.

**Fig. 1:**
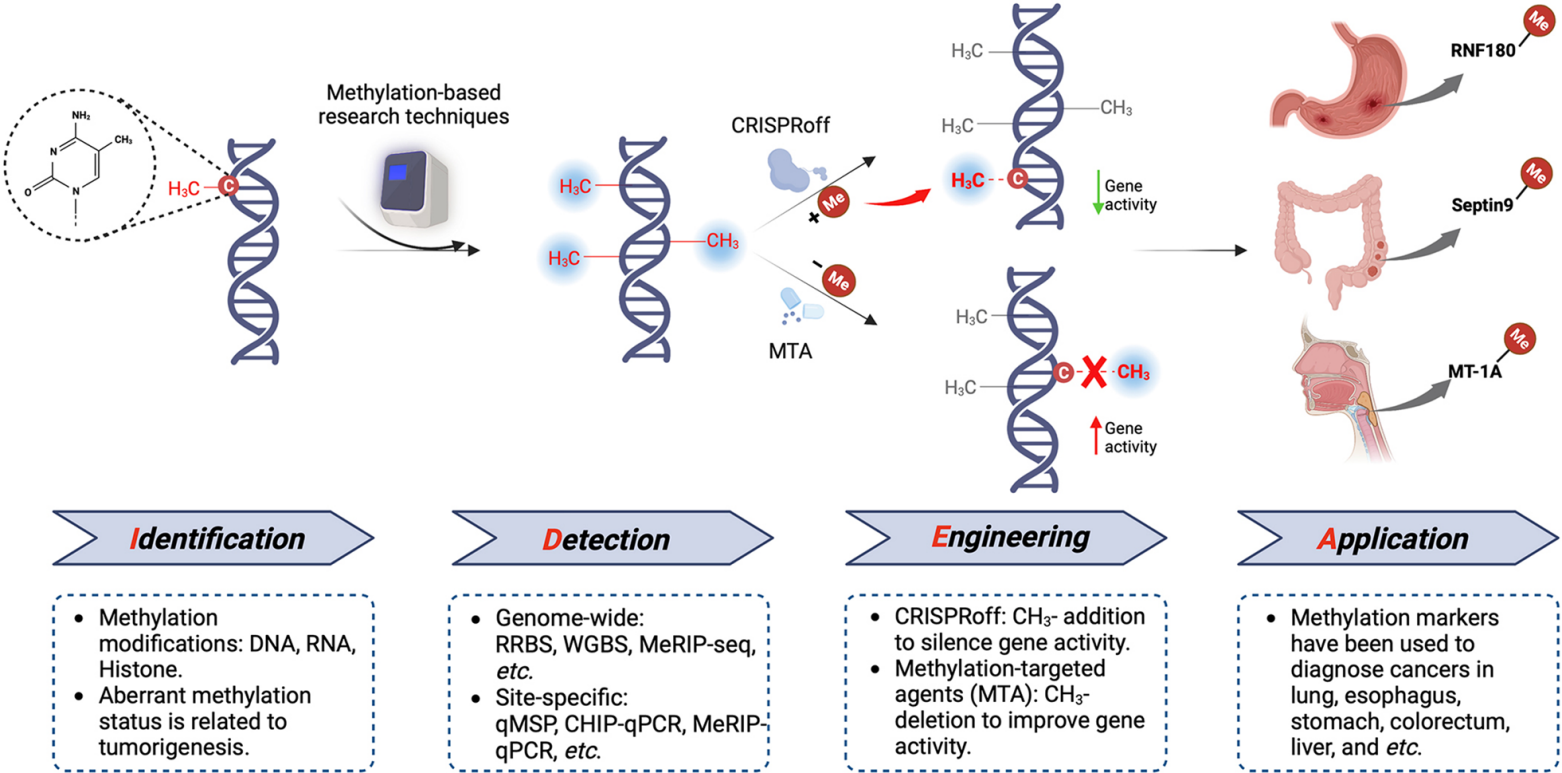
The fundamental theoretical framework “IDEA” of methylation development. The framework for this review is organized into four basic aspects of methylomics research, including (1) *identification* of methylation modifications on various causal genes, RNAs, or chromatin regions and their associated regulatory mechanisms; (2) conventional and advanced methods of methylation *detection* techniques; (3) *engineering* new methods, especially CRISPRoff/on and methylation-targeted agents, for manipulating methylation marks as a possible therapeutic or research strategy; and (4) current and future *application* of methylation markers or methylomics profiling in clinical settings, such as cancer diagnostics or possible future targeted interventions for cancer

In this review, we encapsulate the development of methylation research into four basic stages, including *identification*, *detection*, *engineering*, and *application* (“IDEA”), to build a fundamental theoretical framework (Fig. 1) that: (1) helps establish a basic, comprehensive understanding of the genetic and biochemical basis of the regulatory and mechanistic roles of methylation modifications in cancers; (2) provides readers with a landscape perspective of current innovations in methylation detection and profiling; (3) cover recent efforts to engineer methylation profiling for different cancers and personalized medicine approaches; and (4) summarize advances in methylation detection for clinical diagnostic marker development for various cancers. Approaching methylomics investigation from these four different directions can guide future research addressing both the fundamental biological roles of methylation as well as their development as diagnostic indicators, and eventually potential therapeutic interventions for cancers.

## *Identification*: methylation modifications in tumorigenesis

Methylation marks targeting DNA, RNA, or histones have all been identified as oncogenic drivers through the dysregulation of various biological processes [24]. DNA methylation (DNAm) is one of the most common and well-studied epigenetic modifications, in which a methyl group (CH3) is added to the 5′position of cytosine residues (5-methylcytosine, 5mC) via DNA methyltransferase (DNMT) activity targeting CpG sites [25]. In mammals, most CpG sites in the genome are methylated, including those within gene bodies. The human genome contains about 30,000 CpG islands, 70–80% of which are methylated under physiological conditions [26]. The dynamics of DNAm are mediated by three overarching types of DNA methyltransferase, including those responsible for de novo methylation (e.g., DNMT3A and DNMT3B), those catalyzing demethylation (e.g., TET1, TET2, and TET3), and proteins mediating the duplication of the methylation landscape on newly synthesized strands during DNA replication (e.g., DNMT1 and UHRF1) [27]. Deficiency for a DNMT can cause severe developmental defects resulting in early embryonic lethality [28]. DNA methylation is actively involved in mammalian embryogenesis and is essential in repressing germline-specific genes. Notably, DNA methylation profiles undergo two waves of reprogramming, first during fertilization, then again in primordial germline cell specification [28].

RNA methylation has recently emerged at the frontier of epigenetic research. Unlike DNA and histone methylation, RNA methylation (including mRNA, miRNA, and lncRNA) undergoes a more complex modification process, which can occur post- or co-transcriptionally [29]. Considered the most abundant chemical modification on mRNA and ncRNAs in humans [30], *N*6-methyladenosine (m^6^A) marks are generated by the transfer of a CH_3_ to the N6-position of adenosines in the RRm6ACH (where R = G or A, and H = A, C or U) recognition motif [31]. These marks have been shown to perform essential functions in regulating a wide range of cellular processes, including RNA maturation, transcription, and translation [32]. Similar to DNA and histone methylation, m^6^A modifications are reversible, but are instead coordinated by m^6^A RNA methyltransferase complex (WTAP–METTL3–METTL4–KIAA1429–RBM15) [33, 34] and m^6^A RNA demethylases (FTO, ALKBH5) [31]. Evidence supports that dysregulation of m^6^A enzymes can disrupt biological functions [35], affecting gene expression and cellular differentiation via modulations of various target genes (e.g., circMDK and piRNA-30473) [36, 37] and post-transcriptional RNA-related cellular pathways (e.g., degradation of m^6^A-marked transcripts and mRNA metabolism) [32, 38], inducing the activation of oncogenic signaling pathways to promote cell proliferation, migration, and invasion [37].

Histone methylation is an essential regulatory mechanism in numerous biological processes through control of transcription and replication [39]. The nucleosome structure is comprised of an octameric histone protein complex that includes two dimers (H2A–H2B) and one tetramer (H3–H4) that are wrapped with genomic DNA [40]. Methylation of histone 3 at lysine 4 (H3K4) or lysine 9 (H3K9) are among the most highly conserved and well-studied epigenetic marks; these modifications are catalyzed by histone methyltransferases (HMTs) and removed by histone demethylases (HDMs) [41]. Lysine residues can be mono-, di-, or tri-methylated via lysine-specific HMTs (KMT1A/B/C, 2A, etc.) or demethylated by lysine-specific HDMs (KDM1A/LSD1) [42]. Depending on the position and methylation status, specific histone methyl marks on lysine (K) and arginine (R) residues exert either positive or negative regulatory effects on gene expression [41], with H3K4 (H3K4me3) and K36 (H3K36me3) trimethylation linked to transcriptional activation, and H3K9me2 and K27me3 methylation associated with repression [43]. It should be noted that aberrant regulation of HMT or HDM expression can induce genome-wide alterations in histone methylation status, consequently affecting the expression of oncogenes or tumor-suppressor genes, potentially leading to tumorigenesis [44, 45].

## *Detection*: methylation-based research techniques

**Fig. 2:**
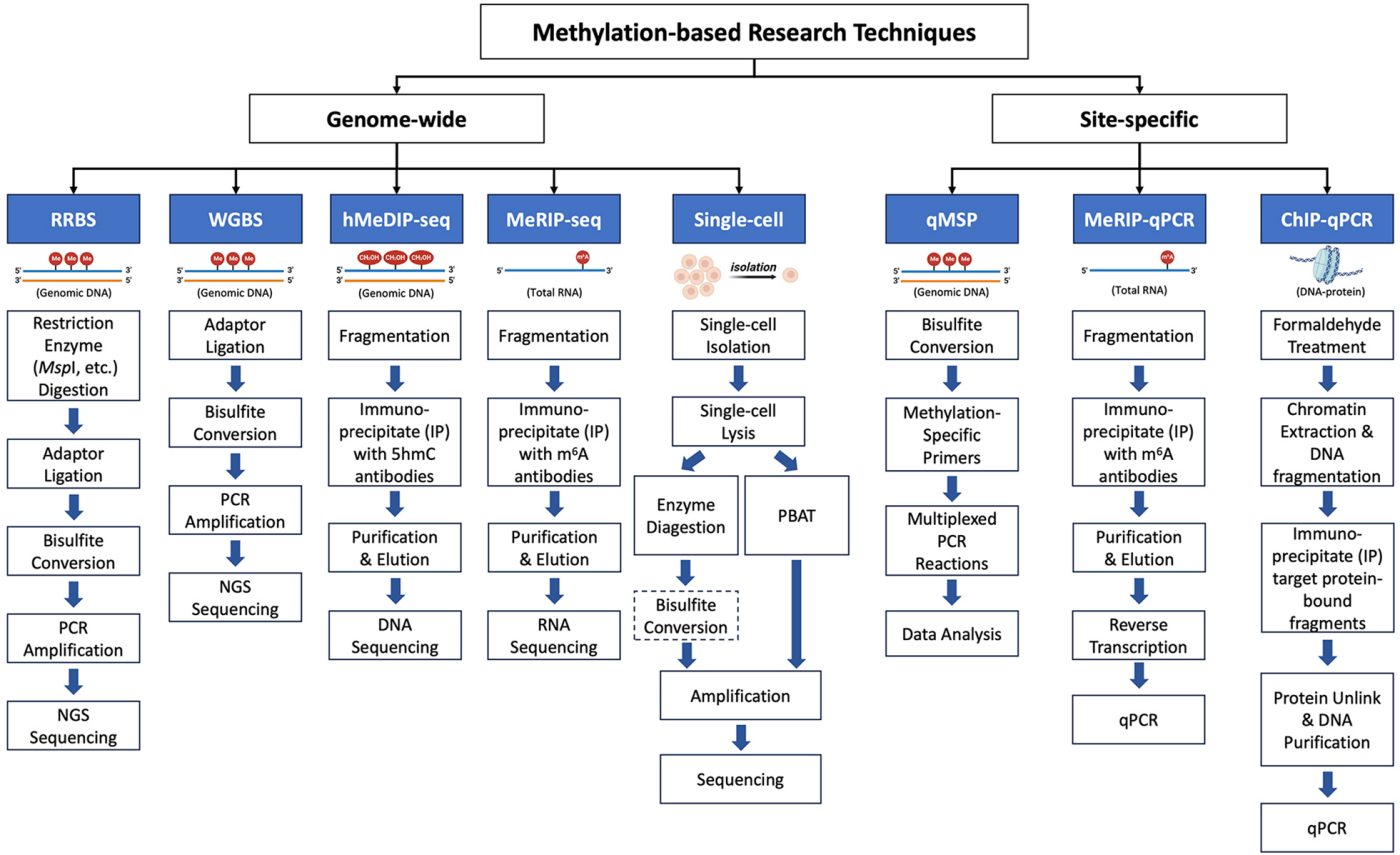
Classification of methylation-based research techniques. The cutting-edge research techniques for studying methylation can be categorized into two groups: *genome-wide* methylomics and *site-specific* methylation detection. The *genome-wide* group mainly includes RRBS, WGBS, hMeDIP-seq, MeRIP-seq, and Single-cell methylation sequencing. The *site-specific* group comprises profiling methods of qMSP, MeRIP-qPCR, and ChIP-qPCR. The input sample type and the schematic of each novel technique are illustrated in the graph

Detecting methylation status has emerged as a highly effective strategy in basic research to understand the mechanisms underlying various processes and pathological conditions, as well as providing valuable clinical diagnostic information. To this end, a wide range of techniques are available for methylation-based research, which can be roughly divided into genome-wide methylomics and site-specific methylation detection. Omics-based techniques include a variety of sequencing technologies, such as reduced representation bisulfite sequencing (RRBS), whole genome bisulfite sequencing (WGBS), hydroxymethylated DNA immunoprecipitation sequencing (hMeDIP-Seq), and methylated RNA immunoprecipitation and deep sequencing (MeRIP-seq). Single-cell methylation sequencing is an omics-based technological breakthrough for characterizing the methylation landscape at single-cell resolution. Site-specific methylation detection mainly comprises quantitative methylation-specific PCR (qMSP), chromatin immunoprecipitation-quantitative real-time PCR (ChIP-qPCR), and methylated RNA binding protein immunoprecipitation-quantitative real-time PCR (MeRIP-qPCR) (Fig. 2).

### Reduced representation bisulfite sequencing (RRBS)

Reduced representation bisulfite sequencing (RRBS) characterizes differential methylation patterns at genome-wide CpG-enriched sites in promoter regions [46]. In this approach, bisulfite sequencing fixes methylation status by sulfide cross-linking, then targets DNA fragments of a specific size range, cut by restriction endonucleases, for sequencing to evaluate DNA methylation levels on CpG islands across the genome [47]. RRBS was introduced and developed by Meissner et al. [48] in 2005, and typically uses the BgIII restriction endonuclease to generate fragments for sequencing. Since then, RRBS has been widely adopted in research and further optimized through higher efficiency MspI to improve CpG enrichment and high throughput sequencing to increase promoter coverage [49]. In 2015, RRBS was first applied to determine DNA methylation status at a single-cell scale [50, 51], facilitating investigations of transcriptional and phenotypic heterogeneity. RRBS is now prevalent in the research and development of cancer and other disease-related biomarkers. For instance, RRBS was used to map DNA methylation profiles in a cohort of 1538 breast cancer patients and subsequently correlate DNA methylation status with tumorigenesis, thus indicating its prognostic potential of these marks [52]. Similarly, RRBS has been used to identify differentially methylated (i.e., differentially regulated) genes in lung cancer [53]. RRBS has also been used to characterize the distinct DNA methylation landscape associated with type 1 diabetes in neonatal umbilical cord blood [54].

Innovations in RRBS will expand its application in complex neurological, autoimmune, and tumor-related diseases. Recent optimization studies have improved genomic coverage and enhanced the extrapolation of transcriptional subtypes, MGMT promoter methylation, and glioma CpG island methylation phenotype for clinical tumor samples [55]. Extended representation bisulfite sequencing, XRBS, was developed as a low-input strategy for targeted DNA methylation sequencing that enables methylation detection on noncoding regulatory elements [56]. This approach uses an optimized MspI enzyme that introduces sample-specific barcodes to expand enhancer and CTCF binding site coverage, increasing the promoter region capture rate [56]. Another variation, double-enzyme RRBS (dRRBS) utilizes MspI with ApekI to increase coverage of CpG sites for better detection of DNA methylation. cfDNA-RBS (cfDNA-reduced representation bisulfite sequencing), a technology that selectively collects DNA at both ends of CCGG sites and employs bisulfite sequencing to discover CG site methylation information at high depth and single-base resolution [57], was developed to overcome the limitations of insufficient coverage in CpG island, enhancer regions, and CTCF binding sites in RRBS. This method is considered the most suitable for investigating DNA methylation biomarkers in tumor research due to its ability to encompass a broader range of gene regulatory regions and its compatibility with small sample sizes [58].

### Whole genome bisulfite sequencing (WGBS)

Whole genome bisulfite sequencing (WGBS) was first proposed by Frommer et al. [59] in 1992 as an innovative approach for mapping methylation profiles at single base resolution, which has become the gold standard approach for detecting DNA methylation. In this technique, unmethylated cytosines in genomic DNA are converted to uracils through bisulfite treatment, while methylated cytosines remain unaffected. Genome-wide methylation levels and sites are subsequently distinguished by whole genome sequencing [59]. Its wide detection range and high-throughput features have led to the use of methylation mapping by WGBS in clinical diagnostic applications for human diseases. For instance, WGBS was used to identify three differentially methylated regions (Dlgap1, TMEM51, and Eif2ak2) in brain tissues of Alzheimer’s disease model mice [60]. Similarly in mice, WGBS was used to establish hydroxymethylation profiles for progressive stages of cervical cancer to screen for potential epigenomic biomarkers [61]. More recently, Magenheim et al. [62] utilized WGBS to obtain methylome profiling in human alveolar and bronchial epithelial tissue. Despite its distinct advantages, such as broad coverage of methylation sites and reduced interference by repetitive regions, SNPs, and other factors [63], WGBS also has drawbacks, most notably its poor accuracy in sequence alignment and low alignment rates. To address these issues, Li et al. [64] developed Guide Positioning Sequencing (GPS) for detecting aberrant DNA methylation, providing improved cytosine coverage (up to 96%) and alignment rates as high as 82.3%, which may lead to its wider adoption in future research. Moreover, as an extension of WGBS, Micro DNA-WGBS has been developed to effectively reduce the amount of sample input required by optimizing the sample processing to reduce genomic DNA degradation, thus improving the library construction and sequencing process [65]. This advancement makes it well-suited for detecting small cell populations or working with limited amounts of DNA samples, such as mammalian preimplantation embryos or minimal human biopsy specimens. Gao et al. [66] successfully utilized this technique to construct a genome-wide methylation map during the development of primate preimplantation embryos using only 100 cells of DNA.

### Hydroxymethylated DNA immunoprecipitation sequencing (hMeDIP-Seq)

hMeDIP-Seq is a method that utilizes immunoprecipitation with 5′-hydroxymethylcytosine (5hmC) antibodies to selectively enrich DNA segments that have undergone hydroxymethylation in the genome. Zhu et al. [67] employed hMeDIP-seq to construct whole-genome DNA methylation and hydroxymethylation profiles of colorectal cancer tissues and their corresponding normal tissues. Qi et al. [68] used hMeDIP-seq to examine the changes in 5hmC that occur during carcinogenesis in the urogenital system, which includes the prostate, urinary tract epithelium, and kidneys. They verified that 5hmC was distributed in urogenital tissues in a tissue-specific manner. hMeDIP-Seq has the advantages of high resolution and whole-genome coverage, making it widely applicable in studying the relationship between hydroxymethylation and diseases.

### Methylated RNA immunoprecipitation and deep sequencing (MeRIP-seq)

*N*6-Methyladenosine (m^6^A) methylation is an essential epigenetic modification in post-transcriptional regulation. This reversible process occurring on mRNAs entails methyl group substitution of the N6 amino hydrogen in adenosines mediated by the METTL3/METTL14 methyltransferase complex [69]. METTL14 was recently identified as a pro-tumorigenic factor in prostate cancer that could potentially serve as a prognostic marker and therapeutic target [70].

Genome-wide profiles of m^6^A sites and methylation levels can be obtained by methylated RNA immunoprecipitation and sequencing (MeRIP-seq). After RNA extraction and fragmentation, m^6^A-specific antibodies are used to capture m^6^A-modified RNA fragments, which are then reverse-transcribed and sequenced [71]. This approach has emerged as a powerful tool for mapping m^6^A-methylated RNA, and is now widely used in research on cancers, cardiovascular diseases, metabolic disorders, and embryonic development. For example, MeRIP-seq was used by Hu et al. [72] to characterize the ALKBH5-PKMYT1-IGF2BP3 regulatory module in gastric cancer metastasis, laying a foundation for further exploration of possible therapeutic targets.

### Single-cell methylation sequencing

Currently, sequencing-based methylation profiling methods typically require a large number of cells per experiment, which consequently hinders studies of rare cell populations and intercellular heterogeneity [73]. However, this obstacle has been largely overcome through advances in single-cell RNA sequencing that enabled epigenomic analysis of diseases at single-cell resolution [74]. Single-cell methylation sequencing, which depends on either restriction digestion [including methylation-insensitive and methylation-sensitive restriction enzymes (MSRE)] or post-bisulfite adaptor tagging (PBAT) [75], has become a critical methylome profiling tool for studying cellular heterogeneity. Its schematic encompasses single-cell isolation (including flow cytometry [76] and microfluidic devices [77]), cell lysis, followed by either the methods of enzyme digestion or PBAT, and subsequently undergoes amplification and sequencing. The enzyme digestion-based single-cell method, such as scRRBS, RSMA, and scTAM-seq, involves the specific recognition and cleavage of DNA strands using restriction endonucleases, e.g., MspI [78], whereas the PBAT profiling technique, such as scWGBS, starts directly with bisulfite conversion, then followed with amplification and sequencing [79]. Smallwood et al. [75] reported single-cell detection of genome-wide methylation levels, reaching CpG coverage of 48.4%, via Single Cell Whole Genome Bisulfite Sequencing (scWGBS), to study embryonic development and tumor heterogeneity in mouse oocytes. Similarly, scWGBS was used to characterize dynamic changes in de novo methylation and demethylation levels at single-cell resolution during early human embryonic development [80]. In addition to scWGBS, single-cell-scale technologies for methylomic profiling such as scRRBS, restriction enzyme-based single-cell methylation assays (RSMA), and single-cell targeted analysis of methylome sequencing (scTAM-seq) have also been used in studies of embryonic development, cellular heterogeneity, pre-implantation genetic diagnosis, and potential cancer therapeutics, among other research topics.

### Quantitative methylation-specific PCR (qMSP)

Quantitative methylation-specific PCR (qMSP) is a fast, cost-effective, sensitive, and specific technique for investigating the methylation status of tumor suppressor genes. This method is based on bisulfite conversion followed by amplification with methylation-specific primers. QMSP has a wide range of clinical applications and is effective in testing aberrant methylation in the early diagnosis of tumors [81], such as methylated Septin9 (mSEPT9) in colorectal cancer or mRNF180 with mSEPT9 in gastric cancer [82]. Similarly, a variant of qMSP was first developed by Song et al., who used RT-qPCR with methylation-specific primers to screen for aberrant methylation patterns at specific methylation sites and validated mSEPT9 as a marker for detecting colorectal cancer in clinical samples [83]. More recently, the combination of methylation and protein markers mRNF180, mSEPT9, and CA724 showed an overall sensitivity of 68.6% for detecting early-stage gastric cancer in a prospective cohort study of 518 patients [82]. QMSP is currently used in clinical, blood-based methylation assays for early cancer diagnosis, and shows potential for further development in prognosis, evaluation of postoperative efficacy, and monitoring of postoperative recurrence.

### Methylated RNA binding protein immunoprecipitation-quantitative real-time PCR (MeRIP-qPCR)

Methylated RNA binding protein immunoprecipitation-quantitative real-time PCR (MeRIP-qPCR) is a variation of MeRIP that incorporates qPCR to measure mRNA methylation levels. In this technique, m^6^A-marked RNAs are immunoprecipitated, purified with m^6^A antibodies, and subjected to reverse transcription and qPCR to assess their methylation status [84, 85]. In work by Liu et al. [84], MeRIP-seq analysis was used to detect m^6^A enrichment near stop codons in HCC versus HCC-adjacent tissues, which revealed CTNNB1 as a target of METTL3-mediated m^6^A modification, then used MeRIP-qPCR to verify CTNNB1 modification under various conditions in subsequent experiments [84]. Furthermore, m^6^A-MeRIP-seq and meRIP-qPCR were used to determine the mechanism by which the METTL3/ZMYM1/E-cadherin signaling pathway promotes epithelial–mesenchymal transition and regulates the metastatic progression in gastric cancer cells [86].

### Chromatin immunoprecipitation-quantitative real-time PCR (ChIP-qPCR)

Chromatin immunoprecipitation-quantitative real-time PCR (ChIP-qPCR) is a well-established method for assessing protein–chromatin interactions at known binding sites, such as transcription factor (TF) binding to DNA. This technique uses antibodies to enrich DNA fragments with histone modifications to determine modification status and quantify TF occupancy at promoter regions [87]. For example, ChIP-qPCR was recently used to study the role of histone lactylation in the metabolic regulation of gene expression by detecting H3K18la enrichment at the YTHDF2 promoter. This analysis showed that the oncogene, YTHDF2, is upregulated by H3K18la modification [88]. Similarly, chromatin immunoprecipitation sequencing (ChIP-seq) can be used to map DNA-binding proteins and histone modifications across the genome at relatively high resolution, and is thus an effective tool for methylation profiling in conjunction with ChIP-qPCR. ChIP-seq analysis of H3K4, H3K36, and H3K79 methylation sites revealed a role of H3K79 methylation in regulating cisplatin resistance in ovarian cancer via C/EBPβ and DOT1L [89].

## *Engineering*: “artificial” methylation for therapeutics and research

Several methods have been developed for manipulating methylation status in vitro and in vivo. Identifying additional methylation markers in tumors may not outweigh the benefits of utilizing methylation as a precise engineering tool to artificially manipulate key genes for clinical purposes. Recent FDA approval of methylation-targeted drugs, along with new strategies for inducing gene silencing or activation through epigenetic editing, especially CRISPRoff/on, may revolutionize targeted methylation-based therapeutic applications in clinic (Table 1).

**Table 1:** Characteristics of “artificial” methylation for the6rapeutics and research

Methods	Methylation modification	Function	Conclusion	References
CRISPRoff/on	Addition/removal of methyl group		• Gene-editing technology, manipulating the methylation landscape • Silencing/activating gene expression • High specificity and durable, long-term, and heritable genetic effects • Anti-tumor/potential therapeutic effects and treatments of neurodegenerative diseases	[90, 91]
Methylation-targeted agents (MTA)	Deletion of methyl group		• 5-Azacytidine and decitabine to treat MDS and AML • Combination treatment with the DNMTi and SGI-110, suggesting strong clinical potential for preventing the recurrence of ovarian cancer • Bidirectional targeting of histone methylation: tazemetostat for treating metastatic or advanced epithelioid sarcoma; CC-90011 for advanced solid tumors or relapsed/refractory marginal zone lymphoma	[92–98]

### The off-targets of clustered regularly interspaced short palindromic repeats gene editing (CRISPRoff)

The Off-targets of Clustered Regularly Interspaced Short Palindromic Repeats (CRISPRoff) is a relatively recent gene-editing technology based on CRISPR-Cas9 that can be used to modulate gene function by manipulating the methylation landscape to alter gene expression without changing genome sequence [90]. Developed by Nuñez and colleagues in 2021 as a programmable methylation editor fusion protein, comprising the *ZNF10* KRAB, Dnmt3A, and Dnmt3L domains with catalytically inactive Cas9 (dCas9), CRISPRoff mediates the methylation of specific DNA sites targeted by a single guide RNA (sgRNA) to silence gene expression [90]. Researchers have made advancements in the development of CRISPRon techniques by leveraging the targeting capability of sgRNA and dCas9 complex, subsequently fusing DNA demethylase to dCas9, thereby facilitating the reversal of the epigenetic gene editing effect associated with CRISPRoff. This innovative approach holds great promise for advancing our understanding of epigenetic regulation and opens up new avenues for manipulating gene expression patterns using the highly versatile CRISPR-Cas9 system [90]. CRISPRoff confers relatively high specificity in silencing genes, even genes lacking CpG islands. Through this targeted DNA methylation, CRISPRoff initiates durable, long-term, and heritable genetic effects that can withstand up to 450 cell passages and stem cell differentiation. Moreover, CRISPRoff was shown to confer pronounced anti-tumor/potential therapeutic effects in induced pluripotent stem cells (ipsCs), HeLa cells (HeLa), human osteosarcoma cells (U2OS), chronic myeloid leukemia cancer cells (K562), and other cells lines [90, 91]. Notably, CRISPRoff was used to suppress the expression of Tau, a microtubule-associated protein linked to Alzheimer’s disease, in vitro, supporting its further exploration for treatments of neurodegenerative diseases [90]. In future and ongoing work, CRISPRoff-mediated silencing activity will undergo further development and optimization, especially in sgRNA targeting, which will inevitably expand its value for research and potential therapeutic application.

### Methylation-targeted agents(MTA)

Epigenetic-based therapies rely on the transcriptional regulatory effects of methylation modifications on DNA, histones, or miRNAs. At present, two types of DNA methyltransferase (DNMT) inhibitors, including 5-azacytidine and decitabine, have been approved by the US FDA to treat myelodysplastic syndromes (MDS) and bone marrow acute myeloid leukemia (AML). 5-Azacytidine and decitabine act on DNA methyltransferases to reduce their catalytic activity, hence hampering the process of DNA methylation, ultimately leading to a slowdown in tumor cell proliferation. It should be noted that these non-specific DNA methylation inhibitors do not directly remove pre-existing methylation modification. Rather, their primary function is to impede further methylation events from taking place [92, 93].

The earliest recognized mechanism of action for these drugs involves competitive inhibition of DNMT binding with DNA, which consequently prevents methylation of the promoter regions of tumor suppressor genes [94]. A phase 3 trial of 472 AML patients ≥ 55 years old who were in remission after induction chemotherapy found that oral azacytidine maintenance therapy could significantly extend overall and relapse-free survival times compared to the placebo, but was accompanied by side effects of gastrointestinal symptoms and neutropenia [95]. Furthermore, combination treatment with the DNMTi and SGI-110 (guadecitabine), the second-generation hypomethylating prodrug of decitabine, can suppress tumorigenesis and promote epigenetic re-sensitization to platinum-based drugs, suggesting strong clinical potential for preventing recurrence of ovarian cancer [96].

Alternatively, bidirectional targeting of histone methylation is another strategy of recent cancer therapies, such as hypomethylation through inhibition of the histone methylase, EZH2, or hypermethylation via inhibition of the histone demethylase, LSD1. Tazemetostat, a first-in-class EZH2 inhibitor, was approved in 2020 to treat metastatic or advanced epithelioid sarcoma, and has been shown to attenuate tumor growth and activate the immune response in bladder cancer [97]. By contrast, CC-90011, the first reversible LSD1 inhibitor, recently entered a first-in-human phase I trial to evaluate its safety, efficacy, and pharmacokinetics in a cohort of 69 patients with advanced solid tumors or relapsed/refractory marginal zone lymphoma. Thus far, reports indicate that the reversible mechanism of CC-90011 confers apparently higher safety compared to that of irreversible LSD1 inhibitors [98].

In addition to these advances in therapeutic targeting of DNA and histone methylation, RNA methyltransferases may also serve as effective therapeutic targets, based on their elevated expression and miRNA methylation levels observed in gastrointestinal cancer cells. Currently, research efforts focusing on miRNA methyltransferases may yield effective therapeutic agents for treating gastrointestinal cancers.

## *Application*: clinical application of methylation in malignancies

Changes in methylation patterns have been shown to silence tumor suppressor genes or activate oncogenes through hyper- or hypomethylation. DNA methylation in the promoter region can prevent the binding of transcription factors, leading to the down-regulation of transcription or gene silencing, whereas genes with hypomethylated promoter regions exhibit increased expression [99]. It is commonly observed that tumor-suppressor genes undergo transcriptional silencing through promoter hypermethylation in cancer [18]. Moreover, studies have revealed significantly higher levels of methylation in benign tissue compared to malignant tissue [100], suggesting that hypermethylated CpG regions are more susceptible to mutations than their normally methylated counterparts [101].

**Fig. 3:**
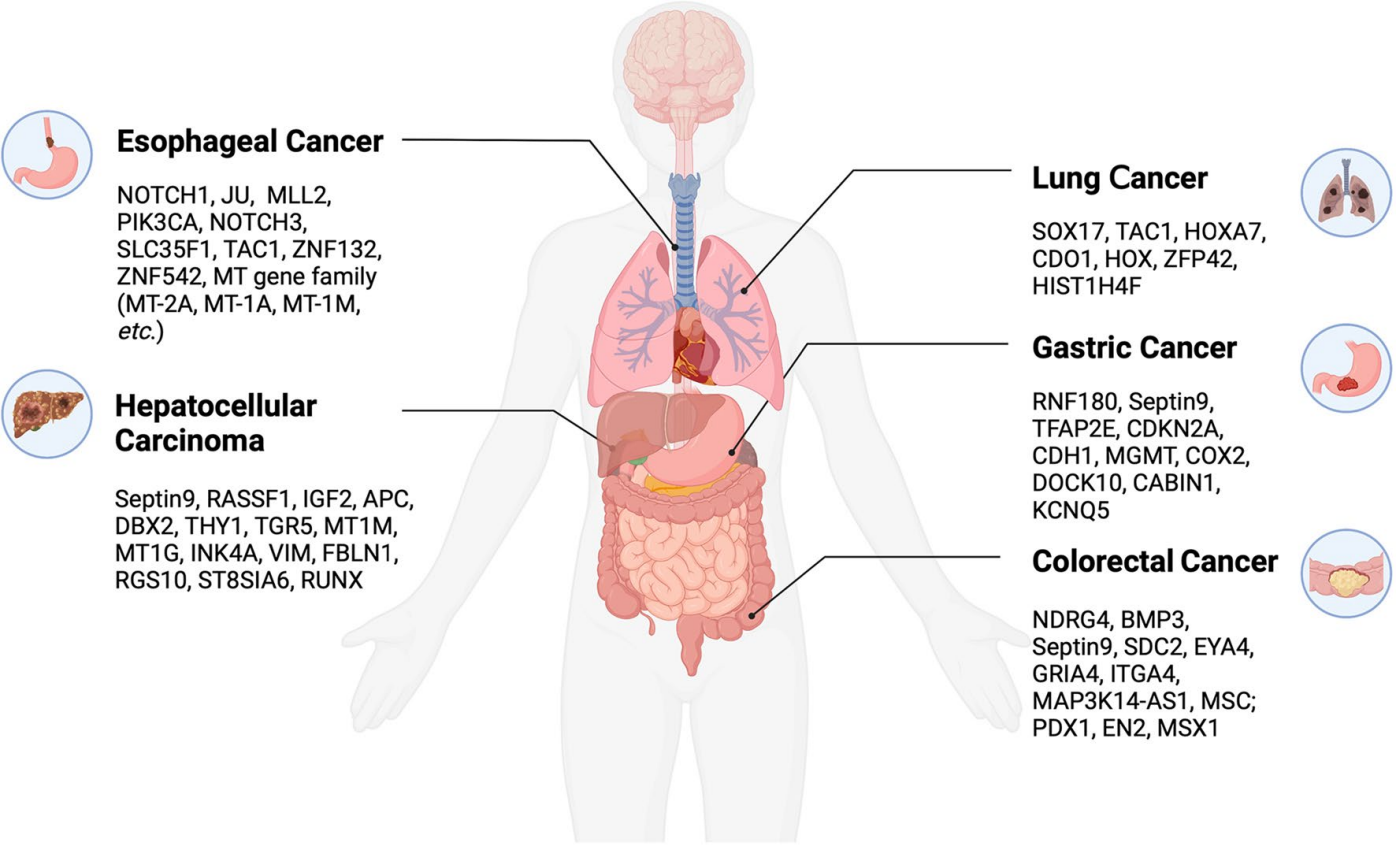
Overview of cancer-associated methylation markers. Several methylation markers have been discovered relevant to a broad range of cancers, such as lung cancer (LC), colorectal cancer (CRC), gastric cancer (GC), hepatocellular carcinoma (HCC), and esophageal cancer (EC)

To date, several methylation markers have been discovered relevant to a broad range of cancers, such as lung cancer (LC), colorectal cancer (CRC), gastric cancer (GC), hepatocellular carcinoma (HCC), and esophageal cancer (EC), although relatively few are adopted for clinical diagnostics. However, recent studies indicate that the potential for DNA methylation markers is steadily growing in in vitro diagnostics and precision medicine through innovations in early detection, diagnosis, and whole-course management of tumors (Fig. 3). Besides, RNA methylation modifications, including 6-methyladenosine (m^6^A), 5-methylcytosine (m^5^C), and 1-methyladenosine (m^1^A), are also closely associated with tumorigenesis, among which m^6^A RNA methylation is the most common and abundant post-transcriptional modification in eukaryotes.

### Lung cancer

Lung cancer (LC) is the leading cause of cancer-related mortality worldwide, accounting for about 1.8 million deaths in 2020 [102]. Although comprehensive treatment regimens, including surgery, chemotherapy, immunotherapy, and targeted therapy, have significantly improved overall survival, prognosis remains relatively poor because the large majority of LC cases are already in the advanced stage when diagnosed, indicating an urgent need for reliable early detection and screening among high-risk individuals. Recent advances in epigenetic biomarkers have made such early screening for LC feasible. High-frequency methylation profiles have been reported for several cancer-specific genes, including SOX17, TAC1, HOXA7, CDO1, HOXA9, and ZFP42, in both preoperative plasma and sputum samples from lymph node-negative stage I and IIA non-small cell lung cancer (NSCLC) patients [103]. Moreover, HIST1H4F, putative universal-cancer-only methylation (UCOM) marker, was found to be hypermethylated in various tumors, including LC, suggesting a role in tumorigenesis in general, as well as a target for early screening and diagnosis [104]. Additionally, the epigenetic regulatory mechanisms of non-coding RNAs and histone modifications have been linked to LC pathogenesis. Chen et al. [105] found that disruption of m^6^A RNA methyltransferase activity by METTL3 due to its SUMOylation can result in oncogenic dysregulation of its target genes. Yuan et al. [106] found that increased histone H3 lysine 36 (H3K36) methyltransferase activity by NSD3, a major 8p11-12 amplicon-associated oncogenic driver, is a crucial regulator of tumorigenesis in lung squamous cell carcinoma (LUSC), but also confers therapeutic susceptibility to bromodomain inhibition [106].

### Colorectal cancer

Colorectal cancer (CRC) is one of the most prevalent malignancies worldwide. In recent years, changes in lifestyles and dietary habits have resulted in a steady increase in the disease burden of CRC [102]. According to *GLOBOCAN 2020, IARC*, CRC is now the third most common cancer and second most common cause of cancer-related death globally [102]. In 2014, the FDA approved a stool-based CRC screening test (Cologuard^®^), which mainly targets the methylation markers, NDRG4 and BMP3 [107]. Among them, NDRG4 is a tumor suppressor gene that inhibits cell proliferation and PI3K-AKT activity, while BMP3 is a growth factor that prevents colorectal tumorigenesis via the ActRIIB/SMAD2 and TAK1/JNK signaling pathways [108, 109]. In most CRC patients, both genes are inactivated due to aberrant hyper-methylation [110], and are therefore attractive diagnostic markers for CRC screening.

Septin9 is a structural protein involved in cytokinesis, and its abnormal methylated status (mSEPT9) often accompanies the early occurrence and development of CRC. Its use as an early diagnostic marker of CRC was validated by QMSP in plasma samples of a cohort of 1031 subjects, with a sensitivity of 76.6% and specificity of 95.9% [111]. In 2016, a plasma-based CRC test targeting Septin9 as the primary methylation marker (Epi proColon^®^) subsequently received FDA approval. In a later study of 184 CRC patients, Bergheim et al. [112] showed that Septin9 methylation had a sensitivity of 84.2% (155/184) in detecting CRC, and that methylation level was related to tumor size, lymph node invasion, and metastasis.

In addition to the above markers, SDC2 methylation (mSDC2) has also been established as an alternative marker for CRC diagnosis that is effective using stool samples. Han et al. [113] reported that a sensitivity and specificity of 90.2% for CRC (stage 0 to IV) diagnosis with mSDC2, although detection rates for advanced and non-advanced adenoma were relatively low at 66.7% and 24.4%, respectively. Using genome-wide methylation microarrays in cfDNA samples of 156 metastatic CRC patients, Barault and colleagues identified five other cancer-specific methylation markers, including EYA4, GRIA4, ITGA4, MAP3K14-AS1, and MSC, that can be informative of tumor burden under different therapeutic regimens [114]. More recently, hypermethylation of PDX1, EN2, and MSX1 were positively linked to CRC progression in RNA-seq analysis, suggesting their potential for development as prognostic markers of CRC [115].

### Gastric cancer

As the fifth most common cancer worldwide, gastric cancer (GC) is a highly heterogeneous complex disease with characteristically high malignancy and poor prognosis that poses a serious threat to human health [102]. Aberrant methylation levels of RNF180 and Septin9 are closely related to GC occurrence, based on observations of increased methylation in clinical plasma samples. RNF180 is an E3 ubiquitin ligase that functions as a tumor suppressor by inhibiting growth, proliferation, and migration in GC cells, but can have pro-tumorigenic effects when hypermethylated [116, 117]. Cheung et al. [118] found irregular hypermethylation of the RNF180 promoter in approximately 56% of GC patients, compared to no RNF180 methylation in healthy controls. Similarly, RNF180 (mRNF180) methylation levels were significantly higher in GC tumor tissues compared with that in adjacent non-tumor tissues [119]. The combination of mRNF180 and mSeptin9 markers can be effective for diagnosing GC. In a prospective cohort study of 518 GC patients, the mRNF180/mSEPTIN9/CA724 marker combination provided a sensitivity of 68.6%, which was markedly higher than that of previously established markers, such as CA19-9, CEA, and CA242, with approximate sensitivities of 20%, 18%, and 10%, respectively [82].

In addition, gastric cancer can be classified into four subtypes based on the TCGA network, including Epstein–Barr virus (EBV)-positive, microsatellite-unstable/instability (MSI), genomically stable (GS), and chromosomal instability (CIN) [120]. Among them, the EBV subtype has characteristically high, aberrant methylation levels, possibly due to the activation of latent promoters during the virus replication cycle in infected cells [121, 122]. Notably, the TFAP2E promoter region is hypermethylated in EBV-positive GC patients; likewise, CDKN2A tumor suppressors are hypermethylated in MSI-associated GC patients [123]. It deserves to be mentioned that abnormal methylation of the CDH1, MGMT, and COX2 genes has also been reported in GC patients with *Helicobacter pylori* infection, while DOCK10, CABIN1, and KCNQ5 methylation levels have also been identified as promising candidate markers for GC screening and surveillance [124]. In addition to DNA methylation, Zhang et al. [125] integrated genomic information from 1938 gastric cancer samples to comprehensively evaluate the m^6^A modification pattern, revealing that the dysregulation of m^6^A regulatory factors plays a crucial role in the occurrence and development of gastric cancer. Zhuo et al. [126] also confirmed that elevated levels of methyltransferase PCIF1, regulated by m^6^A methylation, contribute to the worsening of gastric cancer.

### Esophageal cancer

Esophageal cancer, such as esophageal squamous cell carcinoma (ESCC), is a common digestive tract malignancy, with around 604,000 new cases and 544,000 related deaths reported worldwide in 2020 [102]. Since ESCC has characteristically less severe symptoms in its early stages, most ESCC patients are already in intermediate or advanced stages at the time of diagnosis, which is related to the relatively lower overall 5-year survival rate than that of early-diagnosed cases [127]. However, an increased understanding of the relationship between ESCC tumor development and methylation has enabled gradual improvements in its molecular detection. In particular, hypermethylation of tumor suppressor genes has been broadly documented in EC. For instance, in a Chinese cohort of 91 ESCC patients, Xi et al. [128] found genome-wide aberrant methylation associated with the downregulation of 32 zinc finger family transcription factors. More specifically, 7 hypermethylated CpG sites were detected in the ZNF382 promoter region, in addition to hypermethylation of NOTCH1, JU, MLL2, PIK3CA, and NOTCH3, which together supported the role of abnormal DNA methylation in ESCC occurrence and progression. Similarly, Ma et al. [129] discovered that SLC35F1, TAC1, ZNF132, and ZNF542 were significantly hypermethylated in ESCC, suggesting that methylation of these genes could potentially serve as markers of ESCC diagnosis and monitoring with further validation. In addition, metallothionein (MT) family proteins, such as MT-2A, are highly expressed in cancer-associated fibroblasts, and the methylation of MT-1 genes (MT-1A, MT-1M, and others) is significantly higher in ESCC samples than that in normal esophageal mucosa tissue [130, 131]. Given that the etiology of ESCC remains unclear, the hypermethylation status of tumor suppressor genes may further emerge as an effective strategy for early ESCC diagnosis in the future. Significant progress has been made in treating ESCC through the utilization of RNA methylation. Su et al. [132] discovered that alterations in NSUN2-mediated RNA m5C methylation contributes to the growth of esophageal cancer via LIN28B-dependent stabilization of GRB2 mRNA, which provides a new direction for targeted epigenetic transcriptome therapy in ESCC.

### Hepatocellular carcinoma

Hepatocellular carcinoma (HCC), a prominent histological subtype of primary liver cancer, accounts for more than 80% of all liver cancer cases. Accompanied by high morbidity and mortality rates, HCC is among the prevalent malignancies worldwide and represents the leading cause of death among patients with chronic liver disease [102]. HCC development involves a complex, multi-step process that may include several genetic and/or epigenetic alterations [133]. Oussalah et al. [134] identified a correlation between hypermethylation of the Septin9 promoter region and hepatocarcinogenesis. In addition, some liver cancer-related methylation markers are currently used in clinical research, such as hypermethylation of RASSF1, IGF2, and APC, which can accurately predict poor survival in HCC patients [135]. Another study identified several independently hypermethylated genes in ctDNA collected from HCC patients, including DBX2, THY1, TGR5, MT1M, MT1G, INK4A, VIM, FBLN1, RGS10, ST8SIA6, and RUNX [136]. RNA modifications also play a crucial role in the etiology of HCC. Lan et al. [137] found that the m^6^A methyltransferase complex regulatory element KIAA1429, guided by lncRNA GATA3-AS, selectively methylates and regulates GATA3 pre-mRNA, thereby promoting the proliferation and metastasis of liver cancer cells. This lays the foundation for establishing new molecular diagnostic and therapeutic targets for HCC.

## Discussion

Rapid advances in methylation analysis methods over the past three decades have greatly expanded our understanding of the role of methylation in biological systems, especially cancers. Methylation marks are ubiquitous on genomic DNA, histones, and RNA, and represent a level of transcriptional regulatory information above that in primary DNA sequence. The WGBS-based sequencing platform facilitates the investigation of genome-wide methylation patterns, which is invaluable for characterizing cancer-associated DNA methylation patterns. In contrast, qMSP is a widely used, qPCR-based approach for site-specific detection of methylation status in early-stage tumors. In addition to these research tools, CRISPRoff is a landmark technology for high specificity gene silencing through targeted methylation, which can be used for functional genetic analysis and studies of oncogene regulation and tumorigenesis, and is likely to eventually emerge as an effective therapeutic tool through epigenetic editing.

It should be noted that research focus has gradually shifted from conventional genome-wide methylation detection methods that generate complex, bulk sequencing data from heterogeneous cell populations to simpler, more precise detection techniques. Among these newer strategies, single-cell methylation sequencing can resolve cellular heterogeneity in the tumor microenvironment, enabling mechanistic studies of carcinogenesis at single-cell resolution, and providing a comprehensive perspective of the role of epigenetics in disease development. Furthermore, the combination of PCR-based techniques, high-throughput sequencing, and genome editing can drive the development of molecular diagnostics to improve patient outcomes in clinic.

Several integrative studies have identified differentially expressed genes exhibiting aberrant methylation patterns across multiple types of cancer [138, 139]. The transcriptome directly influences the expression of downstream proliferation-related proteins, as well as various components such as LncRNA, miRNA, circRNA, and other transcriptome biomarkers, which provides a comprehensive understanding of the intricate gene regulatory networks in cancers [140]. However, the complexity of these networks and their dynamic feedback regulation necessitate precise quantitative analysis. Moreover, the instability of RNA poses limitations on the clinical application of transcriptome markers [141]. In contrast, cancer-specific methylation markers exhibit greater sensitivity and stability compared to transcriptome markers, offering promising potential for accurate cancer diagnosis [142]. Aberrant methylation patterns are hallmarks of cancer, and methylation has become an increasingly important feature for cancer research and diagnosis.

Currently, most commercially available DNA methylation assay kits are qPCR-based, and rely on a combination of cancer-specific biomarkers in circulating tumor DNAs in body fluids or other clinical samples. Other methylation detection strategies, such as chips or sequencing platforms that employ large, customized panels of methylation biomarkers, can provide improved diagnostic robustness but at a much higher cost. The implementation of methylation detection technologies in cancer research is still in its early stages, and only a few blood-based tools have been approved for clinical diagnostic application by the FDA or NMPA (in China), such as the Septin9 gene methylation assay for colorectal cancer, the RNF180/Septin9 gene methylation assay for gastric cancer, or the SHOX2/RASSF1A/PTGER4 gene methylation assay for lung cancer. New approaches based on methylation analysis of ctDNA may further improve early detection of various malignancies.

Fundamental research into types of methylation modifications and their diverse physiological and pathological roles, i.e., the *identification* stage, will continue for the foreseeable future, especially as they pertain to cancer development and progression. As *detection* methods improve in accuracy and efficiency, our understanding of the connection between aberrant methylation patterns and oncogenesis will also improve, enabling the refinement and *engineering* of methylation biomarker mapping specific to different cancers and personalized to the genomes of individual patients to accommodate their unique set of risk factors and medical history. Ultimately, this understanding will hopefully drive the *application* of these methylation profiles, or the modifications themselves, as clinical diagnostic tools or advanced personalized interventions for cancer that directly address the regulation of the oncogenes.

## Data Availability

Not applicable.

## Abbreviations

5 mC: 5-Methylcytosine
G: Guanine
m^6^A: *N*6-Methyladenosine
H3K4: Histone H3 lysine K4
H3K9: Histone H3 lysine K9
H3K36: Histone H3 lysine K36
HCC: Hepatocellular carcinoma
CRC: Colorectal cancer
GC: Gastric cancer
EC: Esophageal cancer
ESCC: Esophageal squamous cell carcinoma
LC: Lung cancer
NSCLC: Non-small cell lung cancer
LUSC: Lung squamous cell carcinoma
IDEA: Identification, detection, engineering, application
DNAm: DNA methylation
DNMT: DNA methyltransferase
HMTs: Histone methyltransferases
HDMs: Histone demethylases
RRBS: Reduced representation bisulfite sequencing
WGBS: Whole genome bisulfite sequencing
scWGBS: Single cell whole genome bisulfite sequencing
MeRIP-seq: Methylated RNA immunoprecipitation and deep sequencing
qMSP: Quantitative methylation-specific PCR
ChIP-qPCR: Chromatin immunoprecipitation-quantitative real-time PCR
MeRIP-qPCR: Methylated RNA binding protein immunoprecipitation-quantitative real-time PCR
GPS: Guide positioning sequencing
MSRE: Methylation-sensitive restriction enzymes
PBAT: Post-bisulfite adaptor tagging
TF: Transcription factor
CRISPRoff: The off-targets of clustered regularly interspaced short palindromic repeats
sgRNA: Single guide RNA
ipsCs: Induced pluripotent stem cells
HeLa: HeLa cells
MDS: Myelodysplastic syndromes
AML: Acute myeloid leukemia
UCOM: Universal-cancer-only methylation
MSI: Microsatellite-unstable/instability
GS: Genomically stable
CIN: Chromosomal instability

## References

[1] Ge T, Gu X, Jia R (2022). Crosstalk between metabolic reprogramming and epigenetics in cancer: updates on mechanisms and therapeutic opportunities. Cancer Commun (Lond).

[2] Duan R, Fu Q, Sun Y, Li Q (2022). Epigenetic clock: a promising biomarker and practical tool in aging. Ageing Res Rev.

[3] Ahuja N, Sharma AR, Baylin SB (2016). Epigenetic therapeutics: a new weapon in the war against cancer. Annu Rev Med.

[4] Farooqi AA, Fayyaz S, Poltronieri P, Calin G, Mallardo M (2022). Epigenetic deregulation in cancer: enzyme players and non-coding RNAs. Semin Cancer Biol.

[5] WYATT GR (1950). Occurrence of 5-methylcytosine in nucleic acids. Nature.

[6] Li S, Peng Y, Panchenko AR (2022). DNA methylation: precise modulation of chromatin structure and dynamics. Curr Opin Struct Biol.

[7] Ferragut Cardoso AP, Banerjee M, Nail AN, Lykoudi A, States JC (2021). miRNA dysregulation is an emerging modulator of genomic instability. Semin Cancer Biol.

[8] Cheng Y, He C, Wang M (2019). Targeting epigenetic regulators for cancer therapy: mechanisms and advances in clinical trials. Signal Transduct Target Ther.

[9] Hong M, Tao S, Zhang L (2020). RNA sequencing: new technologies and applications in cancer research. J Hematol Oncol.

[10] Pan S, Chen R (2022). Pathological implication of protein post-translational modifications in cancer. Mol Aspects Med.

[11] Vogelstein B, Papadopoulos N, Velculescu VE, Zhou S, Diaz LA, Kinzler KW (2013). Cancer genome landscapes. Science.

[12] Baylin SB, Jones PA (2011). A decade of exploring the cancer epigenome—biological and translational implications. Nat Rev Cancer.

[13] Chen YC, Gotea V, Margolin G, Elnitski L (2017). Significant associations between driver gene mutations and DNA methylation alterations across many cancer types. PLoS Comput Biol.

[14] Saghafinia S, Mina M, Riggi N, Hanahan D, Ciriello G (2018). Pan-cancer landscape of aberrant DNA methylation across human tumors. Cell Rep.

[15] You JS, Jones PA (2012). Cancer genetics and epigenetics: two sides of the same coin. Cancer Cell.

[16] Shen H, Laird PW (2013). Interplay between the cancer genome and epigenome. Cell.

[17] Li N, Zeng A, Wang Q, Chen M, Zhu S, Song L (2022). Regulatory function of DNA methylation mediated lncRNAs in gastric cancer. Cancer Cell Int.

[18] Widschwendter M, Jones A, Evans I (2018). Epigenome-based cancer risk prediction: rationale, opportunities and challenges. Nat Rev Clin Oncol.

[19] Do C, Dumont E, Salas M (2020). Allele-specific DNA methylation is increased in cancers and its dense mapping in normal plus neoplastic cells increases the yield of disease-associated regulatory SNPs. Genome Biol.

[20] Hernandez-Meza G, von Felden J, Gonzalez-Kozlova EE (2021). DNA methylation profiling of human hepatocarcinogenesis. Hepatology.

[21] Guo J, Zheng J, Zhang H, Tong J (2021). RNA m6A methylation regulators in ovarian cancer. Cancer Cell Int.

[22] Pan J, Liu F, Xiao X (2022). METTL3 promotes colorectal carcinoma progression by regulating the m6A-CRB3-Hippo axis. J Exp Clin Cancer Res.

[23] Hogg SJ, Beavis PA, Dawson MA, Johnstone RW (2020). Targeting the epigenetic regulation of antitumour immunity. Nat Rev Drug Discov.

[24] Dawson MA, Kouzarides T (2012). Cancer epigenetics: from mechanism to therapy. Cell.

[25] Luo H, Wei W, Ye Z, Zheng J, Xu RH (2021). Liquid biopsy of methylation biomarkers in cell-free DNA. Trends Mol Med.

[26] Li E, Zhang Y (2014). DNA methylation in mammals. Cold Spring Harb Perspect Biol.

[27] Dor Y, Cedar H (2018). Principles of DNA methylation and their implications for biology and medicine. Lancet.

[28] Greenberg M, Bourc’his D (2019). The diverse roles of DNA methylation in mammalian development and disease. Nat Rev Mol Cell Biol.

[29] Michalak EM, Burr ML, Bannister AJ, Dawson MA (2019). The roles of DNA, RNA and histone methylation in ageing and cancer. Nat Rev Mol Cell Biol.

[30] Zhou Z, Lv J, Yu H (2020). Mechanism of RNA modification N6-methyladenosine in human cancer. Mol Cancer.

[31] Chen M, Wong CM (2020). The emerging roles of N6-methyladenosine (m6A) deregulation in liver carcinogenesis. Mol Cancer.

[32] Roignant JY, Soller M (2017). M(6)A in mRNA: an ancient mechanism for fine-tuning gene expression. Trends Genet.

[33] Liu J, Yue Y, Han D (2014). A METTL3-METTL14 complex mediates mammalian nuclear RNA N6-adenosine methylation. Nat Chem Biol.

[34] Ping XL, Sun BF, Wang L (2014). Mammalian WTAP is a regulatory subunit of the RNA N6-methyladenosine methyltransferase. Cell Res.

[35] Pendleton KE, Chen B, Liu K (2017). The U6 snRNA m(6)a methyltransferase METTL16 regulates SAM synthetase intron retention. Cell.

[36] Du A, Li S, Zhou Y (2022). M6A-mediated upregulation of circMDK promotes tumorigenesis and acts as a nanotherapeutic target in hepatocellular carcinoma. Mol Cancer.

[37] Han H, Fan G, Song S (2021). piRNA-30473 contributes to tumorigenesis and poor prognosis by regulating m6A RNA methylation in DLBCL. Blood.

[38] Jia G, Fu Y, He C (2013). Reversible RNA adenosine methylation in biological regulation. Trends Genet.

[39] Separovich RJ, Pang C, Wilkins MR (2020). Controlling the controllers: regulation of histone methylation by phosphosignalling. Trends Biochem Sci.

[40] Allis CD, Jenuwein T (2016). The molecular hallmarks of epigenetic control. Nat Rev Genet.

[41] Du J, Johnson LM, Jacobsen SE, Patel DJ (2015). DNA methylation pathways and their crosstalk with histone methylation. Nat Rev Mol Cell Biol.

[42] Black JC, Van Rechem C, Whetstine JR (2012). Histone lysine methylation dynamics: establishment, regulation, and biological impact. Mol Cell.

[43] He K, Cao X, Deng X (2021). Histone methylation in epigenetic regulation and temperature responses. Curr Opin Plant Biol.

[44] Gou D, Liu R, Shan X (2023). Gluconeogenic enzyme PCK1 supports S-adenosylmethionine biosynthesis and promotes H3K9me3 modification to suppress hepatocellular carcinoma progression. J Clin Invest..

[45] Yu SH, Zhu KY, Chen J (2018). JMJD3 facilitates C/EBPβ-centered transcriptional program to exert oncorepressor activity in AML. Nat Commun.

[46] Pan X, Thymann T, Gao F, Sangild PT (2020). Rapid gut adaptation to preterm birth involves feeding-related DNA methylation reprogramming of intestinal genes in pigs. Front Immunol.

[47] Cokus SJ, Feng S, Zhang X (2008). Shotgun bisulphite sequencing of the *Arabidopsis* genome reveals DNA methylation patterning. Nature.

[48] Meissner A, Gnirke A, Bell GW, Ramsahoye B, Lander ES, Jaenisch R (2005). Reduced representation bisulfite sequencing for comparative high-resolution DNA methylation analysis. Nucleic Acids Res.

[49] Ziller MJ, Hansen KD, Meissner A, Aryee MJ (2015). Coverage recommendations for methylation analysis by whole-genome bisulfite sequencing. Nat Methods.

[50] Guo H, Zhu P, Guo F (2015). Profiling DNA methylome landscapes of mammalian cells with single-cell reduced-representation bisulfite sequencing. Nat Protoc.

[51] Hou Y, Guo H, Cao C (2016). Single-cell triple omics sequencing reveals genetic, epigenetic, and transcriptomic heterogeneity in hepatocellular carcinomas. Cell Res.

[52] Batra RN, Lifshitz A, Vidakovic AT (2021). DNA methylation landscapes of 1538 breast cancers reveal a replication-linked clock, epigenomic instability and cis-regulation. Nat Commun.

[53] Sun X, Yi J, Yang J (2021). An integrated epigenomic–transcriptomic landscape of lung cancer reveals novel methylation driver genes of diagnostic and therapeutic relevance. Theranostics.

[54] Laajala E, Kalim UU, Grönroos T (2022). Umbilical cord blood DNA methylation in children who later develop type 1 diabetes. Diabetologia.

[55] Klughammer J, Kiesel B, Roetzer T (2018). The DNA methylation landscape of glioblastoma disease progression shows extensive heterogeneity in time and space. Nat Med.

[56] Shareef SJ, Bevill SM, Raman AT (2021). Extended-representation bisulfite sequencing of gene regulatory elements in multiplexed samples and single cells. Nat Biotechnol.

[57] Liu J, Zhao H, Huang Y (2021). Genome-wide cell-free DNA methylation analyses improve accuracy of non-invasive diagnostic imaging for early-stage breast cancer. Mol Cancer.

[58] Sun HW, Dai SJ, Kong HR (2021). Accurate prediction of acute pancreatitis severity based on genome-wide cell free DNA methylation profiles. Clin Epigenetics.

[59] Frommer M, McDonald LE, Millar DS (1992). A genomic sequencing protocol that yields a positive display of 5-methylcytosine residues in individual DNA strands. Proc Natl Acad Sci U S A.

[60] Zhang S, Qin C, Cao G, Guo L, Feng C, Zhang W (2017). Genome-wide analysis of DNA methylation profiles in a senescence-accelerated mouse prone 8 brain using whole-genome bisulfite sequencing. Bioinformatics.

[61] Han Y, Ji L, Guan Y (2021). An epigenomic landscape of cervical intraepithelial neoplasia and cervical cancer using single-base resolution methylome and hydroxymethylome. Clin Transl Med.

[62] Magenheim J, Rokach A, Peretz A (2022). Universal lung epithelium DNA methylation markers for detection of lung damage in liquid biopsies. Eur Respir J..

[63] Raine A, Manlig E, Wahlberg P, Syvänen AC, Nordlund J (2017). Splinted ligation adapter tagging (SPLAT), a novel library preparation method for whole genome bisulphite sequencing. Nucleic Acids Res.

[64] Li J, Li Y, Li W (2019). Guide positioning sequencing identifies aberrant DNA methylation patterns that alter cell identity and tumor-immune surveillance networks. Genome Res.

[65] Lu H, Yuan Z, Tan T (2015). Improved tagmentation-based whole-genome bisulfite sequencing for input DNA from less than 100 mammalian cells. Epigenomics.

[66] Gao F, Niu Y, Sun YE (2017). De novo DNA methylation during monkey pre-implantation embryogenesis. Cell Res.

[67] Zhu Y, Lu H, Zhang D (2018). Integrated analyses of multi-omics reveal global patterns of methylation and hydroxymethylation and screen the tumor suppressive roles of HADHB in colorectal cancer. Clin Epigenetics.

[68] Qi J, Shi Y, Tan Y (2022). Regional gain and global loss of 5-hydroxymethylcytosine coexist in genitourinary cancers and regulate different oncogenic pathways. Clin Epigenetics.

[69] Sun HL, Zhu AC, Gao Y (2020). Stabilization of ERK-phosphorylated METTL3 by USP5 increases m(6)a methylation. Mol Cell.

[70] Wang Y, Chen J, Gao WQ, Yang R (2022). METTL14 promotes prostate tumorigenesis by inhibiting THBS1 via an m6A-YTHDF2-dependent mechanism. Cell Death Discov.

[71] Saletore Y, Meyer K, Korlach J, Vilfan ID, Jaffrey S, Mason CE (2012). The birth of the epitranscriptome: deciphering the function of RNA modifications. Genome Biol.

[72] Hu Y, Gong C, Li Z (2022). Demethylase ALKBH5 suppresses invasion of gastric cancer via PKMYT1 m6A modification. Mol Cancer.

[73] Farlik M, Sheffield NC, Nuzzo A (2015). Single-cell DNA methylome sequencing and bioinformatic inference of epigenomic cell-state dynamics. Cell Rep.

[74] Sandberg R (2014). Entering the era of single-cell transcriptomics in biology and medicine. Nat Methods.

[75] Smallwood SA, Lee HJ, Angermueller C (2014). Single-cell genome-wide bisulfite sequencing for assessing epigenetic heterogeneity. Nat Methods.

[76] Müller S, Nebe-von-Caron G (2010). Functional single-cell analyses: flow cytometry and cell sorting of microbial populations and communities. FEMS Microbiol Rev.

[77] Shields CW, Reyes CD, López GP (2015). Microfluidic cell sorting: a review of the advances in the separation of cells from debulking to rare cell isolation. Lab Chip.

[78] Liu F, Wang Y, Gu H, Wang X (2023). Technologies and applications of single-cell DNA methylation sequencing. Theranostics.

[79] Miura F, Enomoto Y, Dairiki R, Ito T (2012). Amplification-free whole-genome bisulfite sequencing by post-bisulfite adaptor tagging. Nucleic Acids Res.

[80] Zhu P, Guo H, Ren Y (2018). Single-cell DNA methylome sequencing of human preimplantation embryos. Nat Genet.

[81] Shen SY, Singhania R, Fehringer G (2018). Sensitive tumour detection and classification using plasma cell-free DNA methylomes. Nature.

[82] Xu J, Song J, Wang T (2021). A combination of methylation and protein markers is capable of detecting gastric cancer detection by combined markers. Epigenomics.

[83] Song L, Li Y, Jia J (2016). Algorithm optimization in methylation detection with multiple RT-qPCR. PLoS ONE.

[84] Liu L, Wang J, Sun G (2019). M(6)a mRNA methylation regulates CTNNB1 to promote the proliferation of hepatoblastoma. Mol Cancer.

[85] Yin H, Zhang X, Yang P (2021). RNA m6A methylation orchestrates cancer growth and metastasis via macrophage reprogramming. Nat Commun.

[86] Yue B, Song C, Yang L (2019). METTL3-mediated N6-methyladenosine modification is critical for epithelial–mesenchymal transition and metastasis of gastric cancer. Mol Cancer.

[87] Wu W, Hu Q, Nie E (2017). Hypoxia induces H19 expression through direct and indirect Hif-1α activity, promoting oncogenic effects in glioblastoma. Sci Rep.

[88] Yu J, Chai P, Xie M (2021). Histone lactylation drives oncogenesis by facilitating m(6)a reader protein YTHDF2 expression in ocular melanoma. Genome Biol.

[89] Liu D, Zhang XX, Li MC (2018). C/EBPβ enhances platinum resistance of ovarian cancer cells by reprogramming H3K79 methylation. Nat Commun.

[90] Nuñez JK, Chen J, Pommier GC (2021). Genome-wide programmable transcriptional memory by CRISPR-based epigenome editing. Cell.

[91] Moussa HF, Angstman JF, Khalil AS (2021). Here to stay: writing lasting epigenetic memories. Cell.

[92] Kröger N, Sockel K, Wolschke C (2021). Comparison between 5-azacytidine treatment and allogeneic stem-cell transplantation in elderly patients with advanced MDS according to donor availability (VidazaAllo study). J Clin Oncol.

[93] DiNardo CD, Maiti A, Rausch CR (2020). 10-day decitabine with venetoclax for newly diagnosed intensive chemotherapy ineligible, and relapsed or refractory acute myeloid leukaemia: a single-centre, phase 2 trial. Lancet Haematol.

[94] Kaminskas E, Farrell A, Abraham S (2005). Approval summary: azacitidine for treatment of myelodysplastic syndrome subtypes. Clin Cancer Res.

[95] Wei AH, Döhner H, Pocock C (2020). Oral azacitidine maintenance therapy for acute myeloid leukemia in first remission. N Engl J Med.

[96] Toh TB, Lim JJ, Chow EK (2017). Epigenetics in cancer stem cells. Mol Cancer.

[97] Piunti A, Meghani K, Yu Y (2022). Immune activation is essential for the antitumor activity of EZH2 inhibition in urothelial carcinoma. Sci Adv.

[98] Hollebecque A, Salvagni S, Plummer R (2022). Clinical activity of CC-90011, an oral, potent, and reversible LSD1 inhibitor, in advanced malignancies. Cancer.

[99] Skvortsova K, Stirzaker C, Taberlay P (2019). The DNA methylation landscape in cancer. Essays Biochem.

[100] van Roermund JG, Hinnen KA, Tolman CJ (2011). Periprostatic fat correlates with tumour aggressiveness in prostate cancer patients. BJU Int.

[101] Lin PC, Giannopoulou EG, Park K (2013). Epigenomic alterations in localized and advanced prostate cancer. Neoplasia.

[102] Sung H, Ferlay J, Siegel RL (2021). Global cancer statistics 2020: GLOBOCAN estimates of incidence and mortality worldwide for 36 cancers in 185 countries. CA Cancer J Clin.

[103] Hulbert A, Jusue-Torres I, Stark A (2017). Early detection of lung cancer using DNA promoter hypermethylation in plasma and sputum. Clin Cancer Res.

[104] Dong S, Li W, Wang L (2019). Histone-related genes are hypermethylated in lung cancer and hypermethylated HIST1H4F could serve as a pan-cancer biomarker. Cancer Res.

[105] Chen XY, Zhang J, Zhu JS (2019). The role of m(6)a RNA methylation in human cancer. Mol Cancer.

[106] Yuan G, Flores NM, Hausmann S (2021). Elevated NSD3 histone methylation activity drives squamous cell lung cancer. Nature.

[107] Imperiale TF, Ransohoff DF, Itzkowitz SH (2014). Multitarget stool DNA testing for colorectal-cancer screening. N Engl J Med.

[108] Vaes N, Schonkeren SL, Rademakers G (2021). Loss of enteric neuronal Ndrg4 promotes colorectal cancer via increased release of Nid1 and Fbln2. EMBO Rep.

[109] Zhang Z, She J, Yang J (2018). NDRG4 in gastric cancer determines tumor cell proliferation and clinical outcome. Mol Carcinog.

[110] Wen J, Liu X, Qi Y (2019). BMP3 suppresses colon tumorigenesis via ActRIIB/SMAD2-dependent and TAK1/JNK signaling pathways. J Exp Clin Cancer Res.

[111] Wu D, Zhou G, Jin P (2016). Detection of colorectal cancer using a simplified SEPT9 gene methylation assay is a reliable method for opportunistic screening. J Mol Diagn.

[112] Bergheim J, Semaan A, Gevensleben H (2018). Potential of quantitative SEPT9 and SHOX2 methylation in plasmatic circulating cell-free DNA as auxiliary staging parameter in colorectal cancer: a prospective observational cohort study. Br J Cancer.

[113] Han YD, Oh TJ, Chung TH (2019). Early detection of colorectal cancer based on presence of methylated syndecan-2 (SDC2) in stool DNA. Clin Epigenetics.

[114] Barault L, Amatu A, Siravegna G (2018). Discovery of methylated circulating DNA biomarkers for comprehensive non-invasive monitoring of treatment response in metastatic colorectal cancer. Gut.

[115] Lee Y, Dho SH, Lee J (2022). Hypermethylation of PDX1, EN2, and MSX1 predicts the prognosis of colorectal cancer. Exp Mol Med.

[116] Wang H, Lu Y, Wang M, Wu Y, Wang X, Li Y (2021). Roles of E3 ubiquitin ligases in gastric cancer carcinogenesis and their effects on cisplatin resistance. J Mol Med (Berl).

[117] Sun W, Ma G, Zhang L (2021). DNMT3A-mediated silence in ADAMTS9 expression is restored by RNF180 to inhibit viability and motility in gastric cancer cells. Cell Death Dis.

[118] Cheung KF, Lam CN, Wu K (2012). Characterization of the gene structure, functional significance, and clinical application of RNF180, a novel gene in gastric cancer. Cancer.

[119] Wu Z, Liu H, Sun W (2020). RNF180 mediates STAT3 activity by regulating the expression of RhoC via the proteasomal pathway in gastric cancer cells. Cell Death Dis.

[120] Cancer Genome Atlas Research Network (2014). Comprehensive molecular characterization of gastric adenocarcinoma. Nature.

[121] Matsusaka K, Funata S, Fukuyo M (2017). Epstein–Barr virus infection induces genome-wide de novo DNA methylation in non-neoplastic gastric epithelial cells. J Pathol.

[122] Germi R, Guigue N, Lupo J (2016). Methylation of Epstein–Barr virus Rta promoter in EBV primary infection, reactivation and lymphoproliferation. J Med Virol.

[123] Liu Y, Sethi NS, Hinoue T (2018). Comparative molecular analysis of gastrointestinal adenocarcinomas. Cancer Cell.

[124] Ren J, Lu P, Zhou X (2022). Genome-scale methylation analysis of circulating cell-free DNA in gastric cancer patients. Clin Chem.

[125] Zhang B, Wu Q, Li B, Wang D, Wang L, Zhou YL (2020). M(6)a regulator-mediated methylation modification patterns and tumor microenvironment infiltration characterization in gastric cancer. Mol Cancer.

[126] Zhuo W, Sun M, Wang K (2022). M(6)am methyltransferase PCIF1 is essential for aggressiveness of gastric cancer cells by inhibiting TM9SF1 mRNA translation. Cell Discov.

[127] Miller KD, Siegel RL, Lin CC (2016). Cancer treatment and survivorship statistics, 2016. CA Cancer J Clin.

[128] Xi Y, Lin Y, Guo W (2022). Multi-omic characterization of genome-wide abnormal DNA methylation reveals diagnostic and prognostic markers for esophageal squamous-cell carcinoma. Signal Transduct Target Ther.

[129] Ma K, Kalra A, Tsai HL, Okello S, Cheng Y, Meltzer SJ (2022). Accurate nonendoscopic detection of esophageal squamous cell carcinoma using methylated DNA biomarkers. Gastroenterology.

[130] Shimizu M, Koma YI, Sakamoto H (2021). Metallothionein 2A expression in cancer-associated fibroblasts and cancer cells promotes esophageal squamous cell carcinoma progression. Cancers (Basel).

[131] Krizkova S, Kepinska M, Emri G (2018). An insight into the complex roles of metallothioneins in malignant diseases with emphasis on (sub)isoforms/isoforms and epigenetics phenomena. Pharmacol Ther.

[132] Su J, Wu G, Ye Y (2021). NSUN2-mediated RNA 5-methylcytosine promotes esophageal squamous cell carcinoma progression via LIN28B-dependent GRB2 mRNA stabilization. Oncogene.

[133] Li X, Xu W, Kang W (2018). Genomic analysis of liver cancer unveils novel driver genes and distinct prognostic features. Theranostics.

[134] Oussalah A, Rischer S, Bensenane M (2018). Plasma mSEPT9: a novel circulating cell-free DNA-based epigenetic biomarker to diagnose hepatocellular carcinoma. EBioMedicine.

[135] Villanueva A, Portela A, Sayols S (2015). DNA methylation-based prognosis and epidrivers in hepatocellular carcinoma. Hepatology.

[136] Wu X, Li J, Gassa A (2020). Circulating tumor DNA as an emerging liquid biopsy biomarker for early diagnosis and therapeutic monitoring in hepatocellular carcinoma. Int J Biol Sci.

[137] Lan T, Li H, Zhang D (2019). KIAA1429 contributes to liver cancer progression through N6-methyladenosine-dependent post-transcriptional modification of GATA3. Mol Cancer.

[138] Qureshi SA, Bashir MU, Yaqinuddin A (2010). Utility of DNA methylation markers for diagnosing cancer. Int J Surg.

[139] Farkas SA, Milutin-Gašperov N, Grce M, Nilsson TK (2013). Genome-wide DNA methylation assay reveals novel candidate biomarker genes in cervical cancer. Epigenetics.

[140] Li C, Wang Z, Zhang J (2019). Crosstalk of mRNA, miRNA, lncRNA, and circRNA and their regulatory pattern in pulmonary fibrosis. Mol Ther Nucleic Acids.

[141] Liu Q, Reed M, Zhu H (2022). Epigenome-wide DNA methylation and transcriptome profiling of localized and locally advanced prostate cancer: uncovering new molecular markers. Genomics.

[142] Xu W, Xu M, Wang L (2019). Integrative analysis of DNA methylation and gene expression identified cervical cancer-specific diagnostic biomarkers. Signal Transduct Target Ther.

